# Use of a Novel DNA-Loaded Alginate-Calcium Carbonate Biopolymer Surrogate to Study the Engulfment of Legionella pneumophila by Acanthamoeba polyphaga in Water Systems

**DOI:** 10.1128/spectrum.02210-22

**Published:** 2022-08-11

**Authors:** Sujani Ariyadasa, Craig Billington, Mohamed Shaheen, Nicholas J. Ashbolt, Conan Fee, Liping Pang

**Affiliations:** a Institute of Environmental Science and Research, Christchurch, New Zealand; b School of Biological Sciences, University of Canterbury, Christchurch, New Zealand; c School of Public Health, University of Alberta, Edmonton, Alberta, Canada; d Faculty of Science & Engineering, Southern Cross University, Lismore, New South Wales, Australia; e School of Product Design and Biomolecular Interaction Centre, University of Canterbury, Christchurch, New Zealand; University of Guelph

**Keywords:** amoeba, *Legionella pneumophila*, public health, surrogate, waterborne pathogens

## Abstract

The engulfment of Legionella pneumophila by free-living amoebae (FLA) in engineered water systems (EWS) enhances L. pneumophila persistence and provides a vehicle for rapid replication and increased public health risk. Despite numerous legionellosis outbreaks worldwide, effective tools for studying interactions between L. pneumophila and FLA in EWS are lacking. To address this, we have developed a biopolymer surrogate with a similar size, shape, surface charge, and hydrophobicity to those of stationary-phase L. pneumophila. Parallel experiments were conducted to observe the engulfment of L. pneumophila and the surrogate by Acanthamoeba polyphaga in dechlorinated, filter-sterilised tap water at 30°C for 72 h. Trophozoites engulfed both the surrogate and L. pneumophila, reaching maximum uptake after 2 and 6 h, respectively, but the peak surrogate uptake was ~2-log lower. Expulsion of the engulfed surrogate from *A. polyphaga* was also faster compared to that of L. pneumophila. Confocal laser scanning microscopy confirmed that the surrogate was actively engulfed and maintained within vacuoles for several hours before being expelled. L. pneumophila and surrogate phagocytosis appear to follow similar pathways, suggesting that the surrogate can be developed as a useful tool for studying interactions between L. pneumophila and FLA in EWS.

**IMPORTANCE** The internalization of L. pneumophila within amoebae is a critical component of their life cycle in EWS, as it protects the bacteria from commonly used water disinfectants and provides a niche for their replication. Intracellularly replicated forms of L. pneumophila are also more virulent and resistant to sanitizers. Most importantly, the bacteria’s adaptation to the intracellular environments of amoebae primes them for the infection of human macrophages, posing a significant public health risk in EWS. The significance of our study is that a newly developed L. pneumophila biopolymer surrogate can mimic the L. pneumophila engulfment process in *A. polyphaga*, a free-living amoeba. With further development, the surrogate has the potential to improve the understanding of amoeba-mediated L. pneumophila persistence in EWS and the associated public health risk management.

## INTRODUCTION

Free-living amoebae (FLA) play an important role in the survival and persistence of Legionella pneumophila in engineered water systems (EWS) at ambient temperatures (1). Therefore, an understanding of the interactions between L. pneumophila and FLA is essential to better predicting and controlling the human health risks associated with L. pneumophila in EWS. The intracellular life cycle of L. pneumophila within FLA (trophozoites and cysts), typically in biofilms, facilitates their long-term persistence in EWS, making disinfection challenging (2, 3). The passage of L. pneumophila in modified food vacuoles of FLA also enhances their virulence in human lung macrophages (4).

It is known that the types and the numbers of FLA influence the L. pneumophila population in EWS (5). *Legionella* is frequently associated with the FLA genus *Acanthamoeba* in drinking water distribution systems and in hospital plumbing systems (6). *Acanthamoeba* spp. are the principal hosts for planktonic and biofilm-associated L. pneumophila in EWS, and they make an important contribution to the long-term survival of the bacterium in oligotrophic environments (7). *Legionella* associated with *Acanthamoeba* spp. (principally within cysts) can withstand high water temperatures of 68 to 93°C (8), and *A. polyphaga* encystation of L. pneumophila also increases the chlorine resistance of the bacteria to up to 50 mg/L (9, 10).

Biological and synthetic surrogates have been used to study the interactions of pathogens with FLA in various environmental settings. Feline calicivirus and murine norovirus type 1 were used as surrogates to determine the roles of Acanthamoeba castellanii and Acanthamoeba polyphaga in the environmental persistence and transmission of human noroviruses (11). Likewise, fluorescent polystyrene microspheres have been used to understand pathogen phagocytosis mechanisms in various FLA. Smith et al. (12) observed that both Escherichia coli O157:H7 and 1 μm latex microspheres were engulfed and gradually expelled in the form of fecal pellets by the free-living protist *Tetrahymena.* Additionally, Chrisman et al. (13) reported that A. castellanii trophozoites engulfed and retained both 5.2 μm polystyrene microspheres and heat-inactivated Cryptococcus neoformans fungal cells. Similarly, A. castellanii showed a dose-dependent inhibition in the engulfment of 10 μm polystyrene microspheres and Toxoplasma gondii oocysts in the presence of mannose (14). The disruption of a Dictyostelium discoideum membrane protein gene resulted in a decrease in 1 μm carboxylated polystyrene microsphere and E. coli engulfment (15).

*In situ* investigations of L. pneumophila persistence are essential for the improved prediction and control of L. pneumophila risk in EWS. However, the use of live L. pneumophila is not feasible in many laboratories and field sites due to the need for specialized containment to mitigate the colonization and risk of exposure to research personnel and the public. Therefore, the use of representative and detection-sensitive, nonhazardous surrogates that closely resemble L. pneumophila may be an effective alternative through which to study the interactions and persistence of the bacteria under different environmental conditions.

In our recent study (16), we developed a novel, DNA-loaded, alginate-CaCO_3_ biopolymer surrogate that has a size, shape, cell surface hydrophobicity, and charge similar to those of stationary-phase L. pneumophila, and it displayed similar attachment/detachment behaviors to those of L. pneumophila to/from Pseudomonas fluorescens biofilms grown on stainless-steel in the presence and absence of chlorine in flowthrough bioreactor experiments. The cell surface hydrophobicity and the charge of the surrogate and stationary-phase L. pneumophila were 37.88 (±0.46)%, −21.70 (±0.88) mV and 44.69 (±1.20)%, −27.16 (±0.01) mV, respectively (17). The surrogate’s DNA tracer enables its detection via quantitative real-time polymerase chain reaction (qPCR). Being comprised of food-grade materials, the surrogate can be used in EWS and in eco-sensitive aquatic environments. In this study, we examined whether the new surrogate could mimic L. pneumophila engulfment by *A. polyphaga* trophozoites in dechlorinated, filter-sterilised tap water (DFTW) at 30°C. To the best of our knowledge, this is the first study mimicking *A. polyphaga* engulfment of L. pneumophila using a biopolymer surrogate.

## RESULTS AND DISCUSSION

### Visualisation of surrogate engulfment.

**FIG 1:**
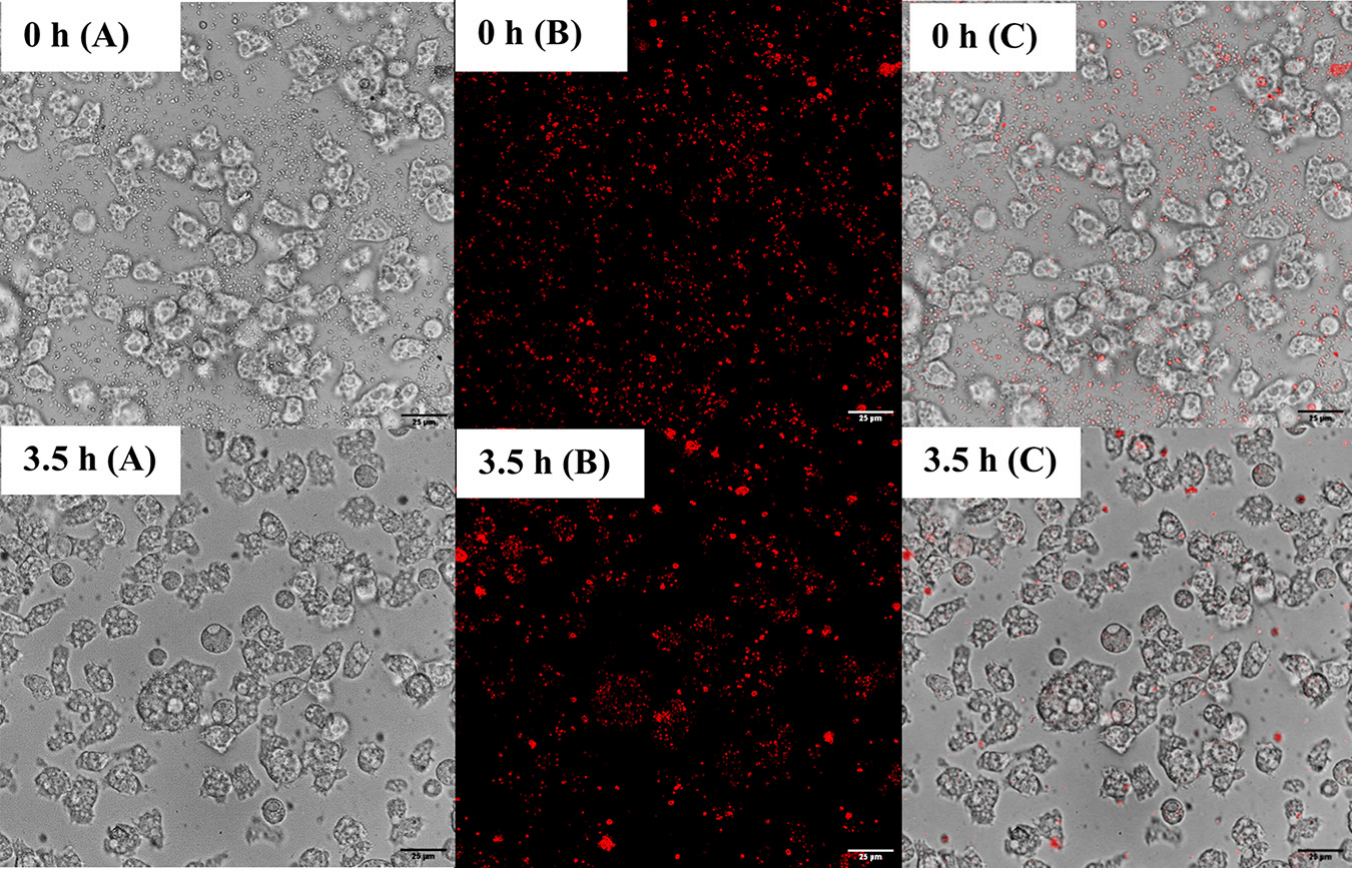
CLSM 45× bright-field (A), fluorescent (B), and composite (C) time-lapse images of fluorescent surrogate coincubated with *A. polyphaga* trophozoites in dechlorinated, filter sterilised tap water at 30°C at 0 (top) and 3.5 (bottom) h of incubation.

Alizarin red-S (ARS) staining enabled the differential detection of surrogate microparticles within the intracellular environment of *A. polyphaga*. Fluorescent surrogate uptake, retention, and expulsion by *A. polyphaga* in DFTW at 30°C were recorded in real time at the single trophozoite level via confocal laser scanning microscopy (CLSM) time-lapse imaging. The formation of acanthopodia and the engulfment of the surrogate microparticles by the trophozoites were observed immediately after coincubation (Fig. S1). Surrogate microparticles were observed outside the trophozoites at 0 h (Fig. 1A, B, and C). However, after 3.5 h, surrogate microparticles were almost exclusively found within the trophozoites, concentrated within both the cytosol and intracellular vacuoles (Fig. 1A, B, and C). The trophozoites were also less motile at 3.5 h than at 0 h (see supplementary videos 1 and 2).

**FIG 2:**
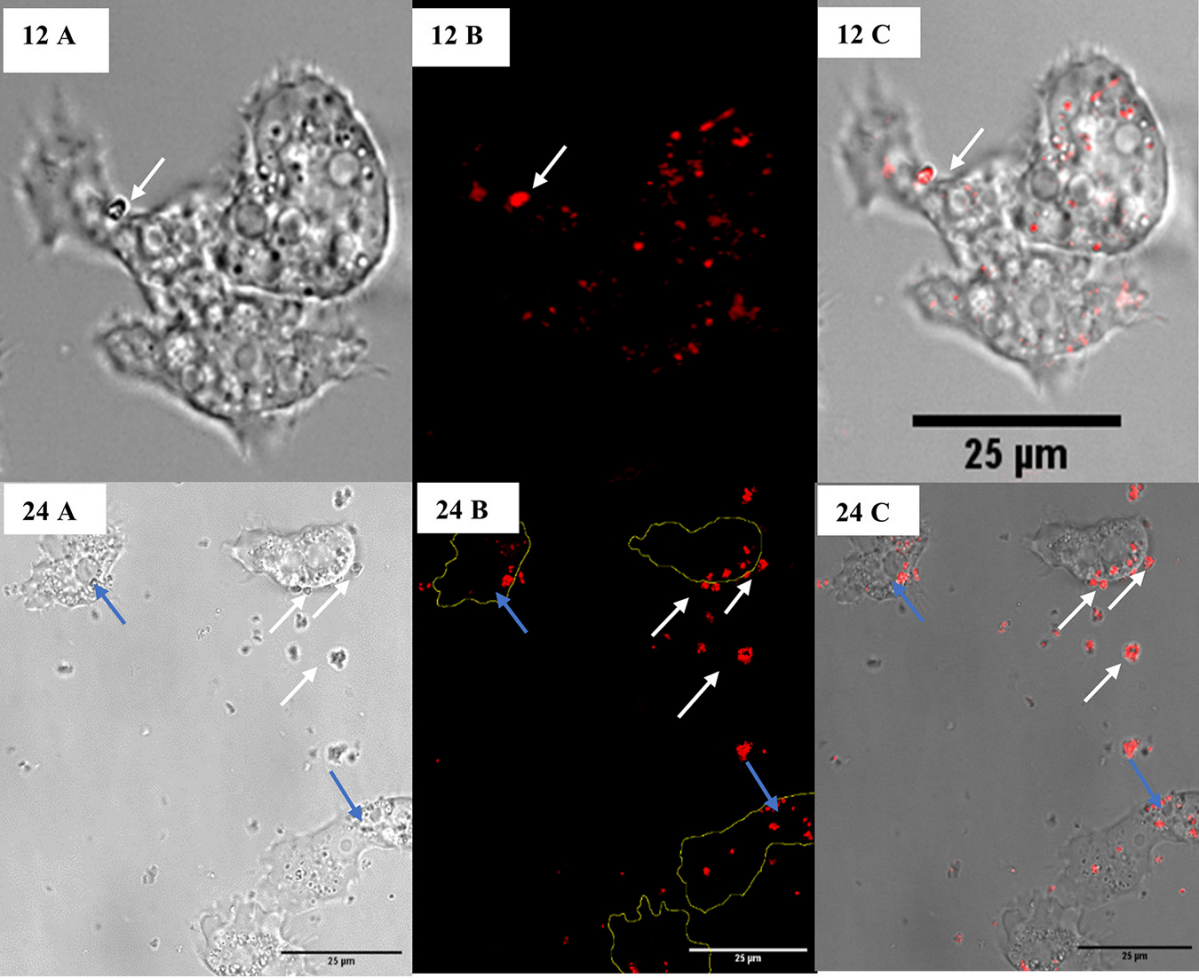
CLSM bright-field (A), fluorescent (B), and composite (C) images of surrogate exocytosis by *A. polyphaga* trophozoites at 12 h at 100× (top) and at 24 h at 60× (bottom). The white arrow points to a clump of surrogate microparticles being exocytosed by a trophozoite, and the blue arrows point to retained surrogate microparticles that are concentrated at the periphery of the trophozoite membranes.

The expulsion of surrogate microparticles was observed after 12 h of coincubation. Motile trophozoites discharged surrogate clumps to the medium through exocytosis (Fig. 2, top row). An analysis of the images showed an average of 2.9 (±2.4) extracellular surrogate clumps per field of view. The proportion of trophozoites releasing engulfed surrogate microparticles was not able to be calculated due to their motility. However, the majority of the trophozoites retained brightly fluorescent surrogate microparticles and were observed to be moving using cytoplasmic projections (acanthopodia, supplementary video 3). A proportion of the trophozoites containing surrogate microparticles were observed to have retracted their acanthopodia and formed a round shape (Fig. S2). These rounded trophozoites appeared to be nonmotile compared to those with acanthopodia. The average ratio of trophozoites with acanthopodia to rounded trophozoites per field of view was 5 (±1.6):1 (Table S1). Untreated amoeba samples with no surrogate microparticles showed no trophozoite rounding (observed at 12 h). Therefore, the change in trophozoite morphology appeared to be associated with the engulfment and retention of the surrogate microparticles. Grazing-induced alteration in amoeba morphology has previously been observed in A. proteus trophozoites fed with bacteria (18). The results of that study showed that the morphology alteration in trophozoites fed with pathogenic bacteria was significantly higher than that of those fed with nonpathogenic bacteria. The authors suggested that the prevalence of rounded cells may control the size of the trophozoites’ population by restricting their ability to “hunt” for food material. Another study indicated that the acanthopodia retraction and cell rounding may be associated with trophozoite encystment (19).

Over the next 12 h, the average number of extracellular surrogate clumps in the medium increased significantly (*P* < 0.05) and reached 39.0 (±9.0) per field of view, indicating an increase in surrogate exocytosis by the trophozoites. An analysis of the sizes of the expelled clumps via surface area measurement (Fig. S3) showed that most (60%) of the clumps were small (<1 μm^2^), while 37% were between 1 to 3 μm^2^. A smaller percentage (3%) of large (3 to 5 μm^2^) clumps was also observed. The average surface area of these extracellular surrogate clumps was 1.1 (±0.8) μm^2^. The average surface area of the surrogate introduced into the experiment was 2.3 (±0.7) μm^2^. Therefore, it is likely that the clumps of size <1 μm^2^ were a result of the partial digestion of surrogate microparticles. Measurements of the expelled surrogate microparticles are presented in a surface area basis rather than one of volume, as only a 2-D image analysis was possible with our microscopy setup.

Extracellular surrogate microparticles remained in close proximity to the trophozoites after expulsion (Fig. 2, bottom row). It is not known why this was observed. However, one possible explanation is that partial degradation of the surrogate may have occurred within the trophozoites, releasing biopolymer fragments from the microparticle surfaces and causing partial attachment to the cell following exocytosis. Surrogate microparticles retained in the trophozoites were concentrated toward the periphery of the cell membrane prior to their release into the medium (Fig. 2, bottom row). The accumulation of indigestible material near the cell membrane is known to facilitate exocytosis in amoebae (20).

**FIG 3:**
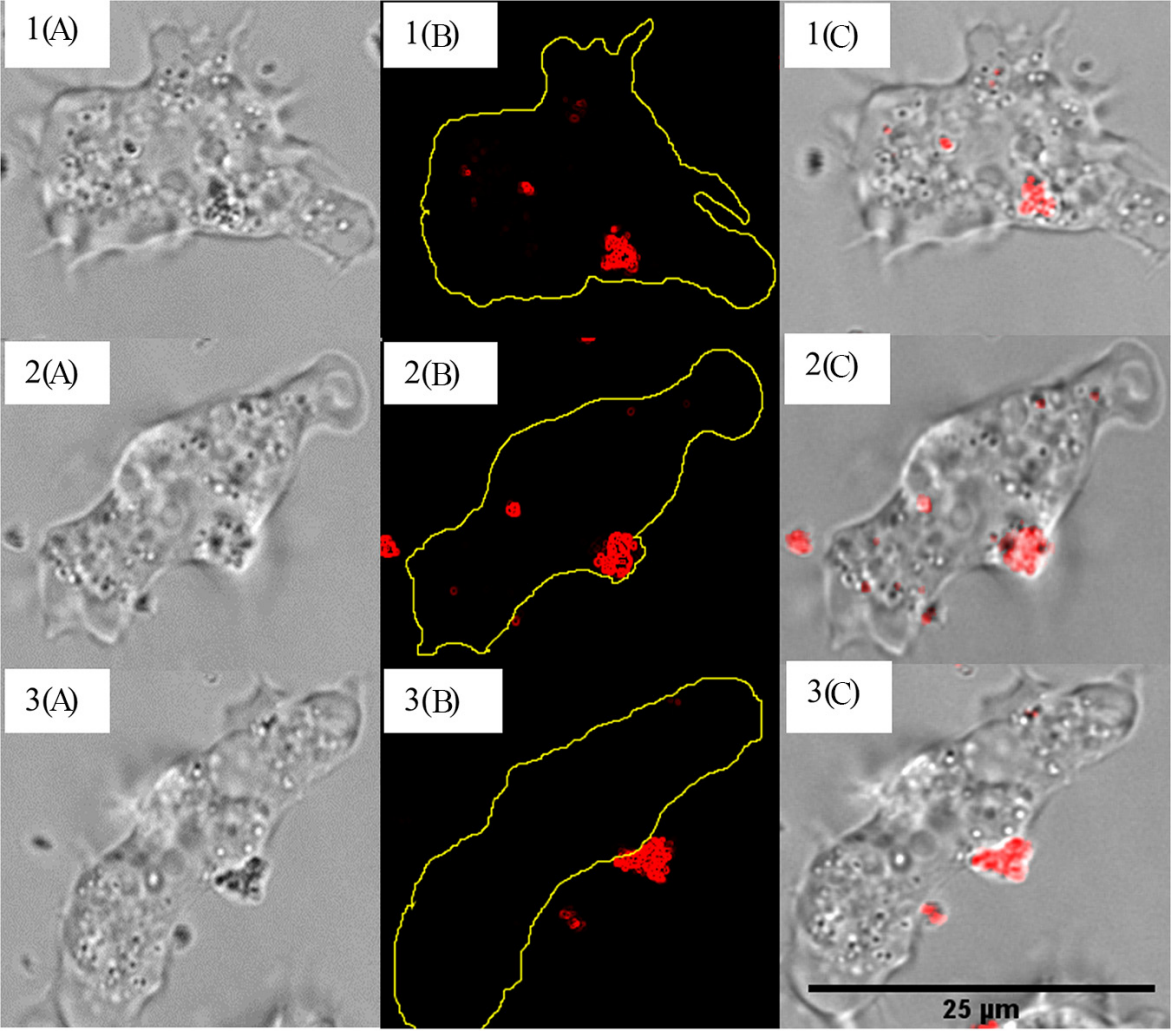
CLSM 100× bright-field (A), fluorescent (B), and composite (C) images of an *A. polyphaga* trophozoite releasing a surrogate clump at 48 h of coincubation.

After 48 h, increased surrogate exocytosis by the trophozoites was observed. The trophozoites expelled large aggregates of surrogate microparticles or vesicles (Fig. 3), with only a few surrogate microparticles remaining within the trophozoites. Compared to the observations at 24 h, the average number (73.9 ± 9.5 per field) and surface area (2.5 ± 2 μm^2^) of the extracellular surrogate microparticles increased significantly (*P* < 0.05). The presence of medium (1 to 3 μm^2^) and large (3 to 5 μm^2^) surrogate clumps also increased by 38% and 12.7%, respectively (Fig. S3). Extracellular surrogate clumps of size >5 μm^2^ were not observed previously but accounted for 9.3% of the clumps at 48 h. Despite the increased exocytosis, no significant variation was observed in the ratio of motile trophozoites with acanthopodia to rounded trophozoites (*P* > 0.05) (Table S1). However, lysed trophozoites were observed for the first time at 48 h (Fig. S4; supplementary video 4). It is inconclusive whether surrogate engulfment and retention contributed to trophozoite lysis.

We were unable to visualize the live L. pneumophila engulfment due to the biohazard risk management rules for our microscopy facility. Thus, we used qPCR for comparison studies. The use of heat-fixed or nonpathogenic strains of L. pneumophila for visualization experiments was considered to reduce the biohazard risk, but these approaches were not pursued due to the inability of the fixed bacteria to produce signals that may be important in engulfment (21) and the potential differences in cell surface properties of noninvasive L. pneumophila strains, such as dotA− (22).

### Quantification of surrogate and L. pneumophila engulfment by *A. polyphaga*.

**FIG 4:**
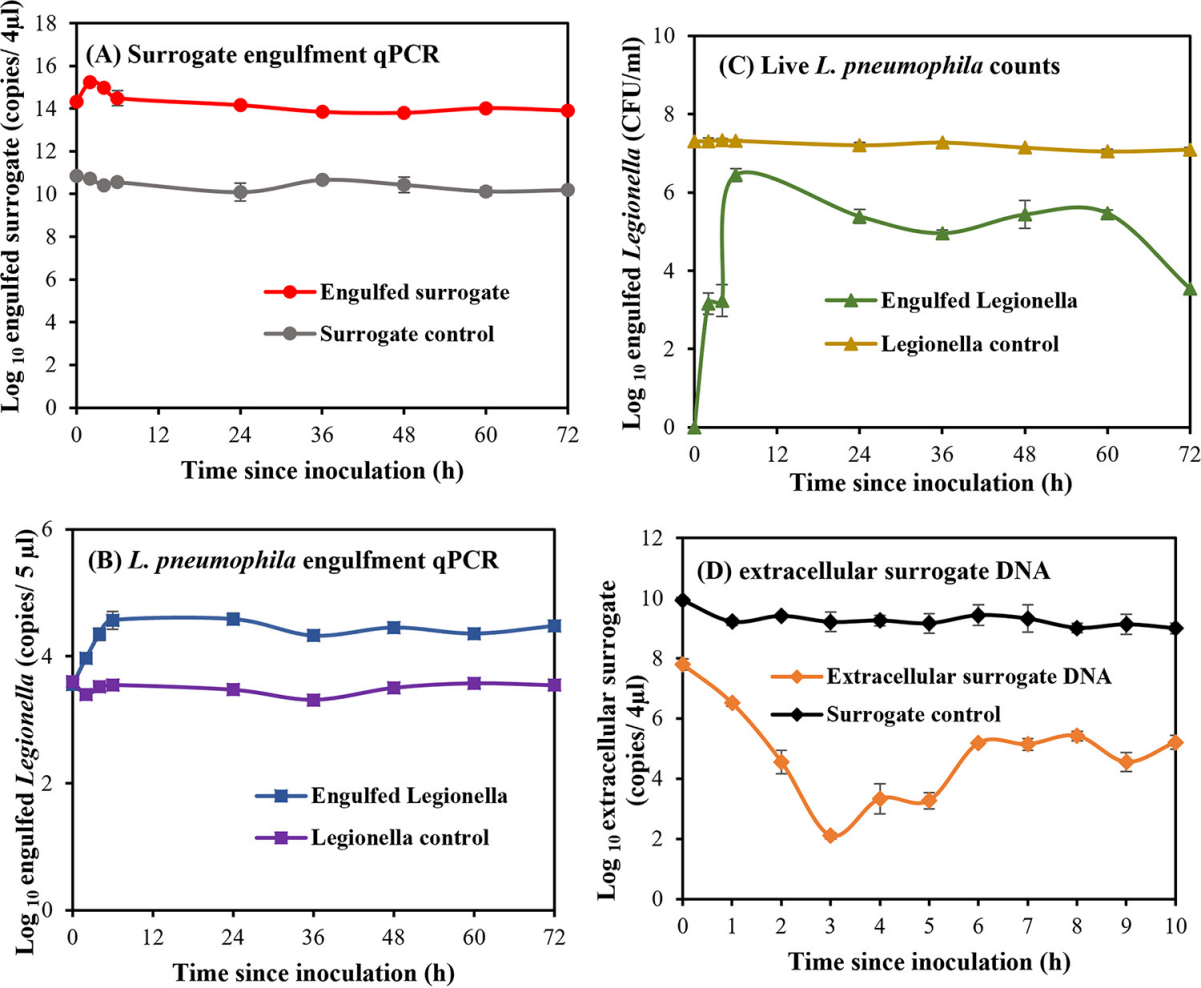
The log-transformed qPCR concentrations of engulfed surrogate tracer DNA, L. pneumophila DNA, and nonstained extracellular (nonengulfed) surrogate tracer DNA (A ●, B ■, and D ♦, respectively) in surrogate-*A. polyphaga* and L. pneumophila*-A. polyphaga* cocultures at 30°C. The counts of live L. pneumophila engulfed by *A. polyphaga* are shown in panel C (▴). The averages of 6 sample replicates are reported, and the error bars represent the standard deviation at each sampling point.

Concentrations of the intracellular surrogate and L. pneumophila were measured by qPCR after 72 h at 30°C in parallel experiments to determine their engulfment kinetics by *A. polyphaga* trophozoites. The surrogate’s DNA tracer was detected in the trophozoites, even at time zero, suggesting that the surrogate was engulfed immediately after the coincubation (Fig. 4A). The concentration of intracellular surrogate (C_S_) rose quickly by 1 log and then peaked after 2 h of coincubation. The earliest surrogate expulsion was observed immediately after this peak engulfment, as indicated by a 1 log reduction in C_S_ over the next 4 h. The rate of exocytosis declined after this initial burst, with only a 0.45 log reduction observed from 6 to 72 h. However, the reduction of C_S_ from 2 h to 72 h post-coincubation was significant (*P* < 0.05). The concentration of surrogate DNA in the control samples remained largely unchanged at ~11 log throughout the experiment.

Similar to the surrogate, DNA from engulfed L. pneumophila (C*_Lp_*) was detected at time zero (Fig. 4B). However, C*_Lp_* showed a slow, gradual increase compared to C_S_, reaching peak engulfment after 6 h of coincubation with a significant 1 log increase in copies (*P* < 0.05). C*_Lp_* showed no significant change (*P* > 0.05) from the time of peak engulfment to 24 h, suggesting a pause in L. pneumophila engulfment. This observation agrees with that of Bowers and Olszewski (20), who showed that the membrane ingestion rates of trophozoites may reach zero upon their saturation with food material. A significant decrease (*P* < 0.05) in C*_Lp_* was observed over the remainder of the experimental period (72 h), as confirmed by qPCR and colony counts, indicating the digestion and/or exocytosis of the internalized L. pneumophila. It is also likely that the release of internalized L. pneumophila through trophozoite lysis also contributed toward the observed decrease in C*_Lp_* at 72 h. We presume that this may have resulted in the loss of L. pneumophila cells before the trophozoites were harvested for DNA extraction and qPCR. A previous coculture study conducted using the same strains of L. pneumophila and *A. polyphaga* reported that the bacteria displayed cytopathogenicity toward the amoebae after long coincubations, resulting in a 38.3% decrease in live trophozoites at 72 h (23).

Engulfed L. pneumophila were also enumerated by culture at the same time intervals as the qPCR assay, (Fig. 4C). However, no colonies of intracellular L. pneumophila were recovered at 0 h. This may be due to the better sensitivity of the qPCR techniques, compared to that of the culture method, at low concentrations of the bacteria. Previous studies have reported higher detectable levels of *Legionella* when using qPCR, compared to the culture method (24). The viable intracellular L. pneumophila counts increased from 3.2 log CFU/mL at 2 h to 6.5 log CFU/mL at 6 h of coincubation. The maximum viable bacterial counts were obtained at 6 h, suggesting peak engulfment, and being in concordance with the qPCR results. However, a significant (*P* < 0.05) decrease in counts was observed from 6 h to 24 h, in contrast to the qPCR results. This difference could be explained by a fraction of the engulfed L. pneumophila being digested by trophozoites during this time. A further, statistically significant (*P* < 0.05) 2.9 log CFU/mL net decrease in viable counts was observed from 24 h to 72 h, suggesting digestion and/or progressive exocytosis. The bacterial counts observed are consistent with those reported by Patrizia et al. (23), who reported similar reductions of viable L. pneumophila strain Philadelphia 1 that were engulfed by *A. polyphaga* at 30°C. According to their results, a 1 log CFU/well overall decrease was observed for this strain of L. pneumophila, whereas other environmental strains of the bacteria, such as the serogroups 1, 6, and 9, showed an approximately 1 to 2 log CFU/well increase in their viable cell numbers when engulfed by *A. polyphaga.* Furthermore, no significant reduction (*P* > 0.05) was observed ihen t L. pneumophila control samples (no trophozoites) in our study after a 0.2 M HCl-KCl buffer treatment under the same conditions.

In addition to measuring the concentrations of intracellular surrogate and L. pneumophila, the concentrations of extracellular surrogate DNA (C_exs_) were quantified (Fig. 4D). C_exs_ had an approximately 2 log decrease after 1 h of coincubation, suggesting that the surrogate was readily engulfed by the trophozoites. A further 4 log reduction was observed over the next 1 to 3 h as the surrogate engulfment progressed. The lowest C_exs_ was recorded at 3 h of coinoculation (2 log copies) and reflected a peak in surrogate engulfment by the trophozoites. The time taken for the trophozoites to reach maximum surrogate engulfment was similar to that observed in the CLSM experiments at 3 and 3.5 h, respectively. Therefore, it appears that the staining of the surrogate with ARS did not affect the surrogate engulfment activity by the trophozoites. C_exs_ then increased by 4 log from 3 h to 6 h of coincubation, suggesting exocytosis of the engulfed surrogate. As such, the surrogate was exocytosed soon after peak engulfment. C_exs_ remained largely unchanged over the next 6 to 10 h. The concentration of surrogate DNA in the control samples, which were lacking trophozoites, remained constant throughout the experiment.

**FIG 5:**
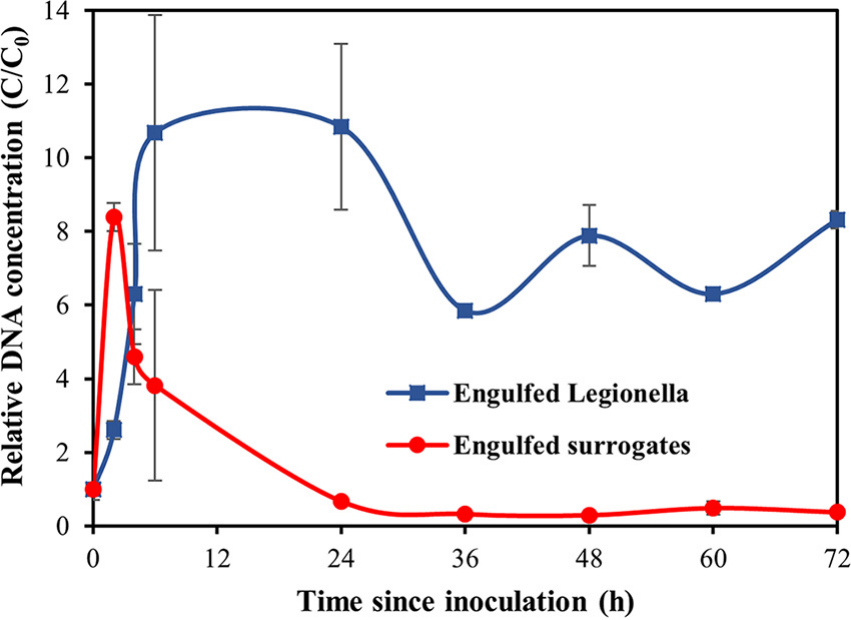
Normalized concentrations (C/C0) of the surrogate (●) and L. pneumophila (■) engulfment kinetics of *A. polyphaga* trophozoites in dechlorinated, filter-sterilised tap water at 30°C over a period of 72 h. Error bars represent the normalized standard deviation of 6 sample replicates.

To compare the engulfment kinetics of the surrogate and L. pneumophila, their log-transformed qPCR concentrations were normalized by the log-transformed qPCR concentrations at 0 h (C/C_0S_ and C/C_0_*_Lp_*, respectively). As indicated in Fig. 5, both the surrogate and the bacteria were engulfed from time zero, immediately after the cocultures were established. However, the surrogate was engulfed at a higher rate than were the bacteria, indicated by the greater slope of the C/C_0S_. As a result, the trophozoites were saturated with engulfed surrogate microparticles as early as 2 h into the coincubation. In contrast, the L. pneumophila engulfment had a more gradual increase, and maximum engulfment was observed after 6 h of coculture. The magnitude of C/C_0S_ at peak engulfment was about 2 log lower than the magnitude of C/C_0_*_Lp_* (8.4 versus 10.83, respectively) (Fig. 5). It appeared that there was an initial preference for the engulfment of the biopolymer surrogate over L. pneumophila, which is supported by evidence that FLA may preferentially prey upon non-L. pneumophila cells using a yet to be identified recognition system (25). Despite this, the gradual increase in C/C_0S_ and C/C_0_*_Lp_* from time zero to peak engulfment suggests that the trend in surrogate phagocytosis may be similar overall to that of L. pneumophila. However, further studies using lower ratios of amoeba to *Legionella* and amoeba to surrogate are required to determine this conclusively.

Following saturation, the trophozoites containing engulfed L. pneumophila had a window of feeding inactivity with no engulfment or exocytosis, whereas the trophozoites containing engulfed surrogate commenced exocytosis immediately. Surrogate exocytosis began at 2 h, whereas L. pneumophila exocytosis had a delayed onset until 24 h. Exocytosis resulted in the loss of most of the engulfed surrogate by 72 h, while a large fraction of the L. pneumophila remained in the trophozoites at this time. The log-transformed average qPCR concentrations of surrogate and L. pneumophila control samples with no trophozoites remained largely unchanged at a value of 1.0 throughout the experiment.

Our findings indicate the potential of biopolymer surrogates as a tool for mimicking L. pneumophila engulfment by *A. polyphaga* in EWS. The internalized and nonreplicating surrogate microparticles represented a good comparator for the bacterial engulfment kinetics by *A. polyphaga* in the scope of this study, as *A. polyphaga* does not permit the intracellular replication of L. pneumophila (5, 23). With further validations under more EWS-representative conditions, such as different amoeba to bacteria ratios, different temperatures and amoeba hosts, the presence of drinking water biofilms, and the presence/absence of disinfectants, the new surrogate may be used as a promising approach by which to provide new insights into the amoeba-mediated persistence of L. pneumophila in water systems. However, as a nonbiological entity, the new biopolymer surrogate will not be able to fully mimic the features of interactions between L. pneumophila and protozoa, such as intracellular replication and the production of signalling molecules within biofilms.

## MATERIALS AND METHODS

### Microbial strains and culture conditions.

L. pneumophila serogroup 1 strain Philadelphia, ATCC 33152, and *A. polyphaga*, ATCC 30461, were obtained from the American Type Culture Collection (ATCC), USA. Stationary-phase L. pneumophila cell suspensions were established according to Ariyadasa et al. (17).

*A. polyphaga* axenic cultivation was conducted following the ATCC protocol (“Acanthamoeba polyphaga [Puschkarew] Page | ATCC,” 2021). Trophozoites were cultured in a T-25 tissue culture flask (Nunc EasYFlask, Thermo Fisher Scientific, USA) that contained 5 mL of sterile peptone yeast extract glucose (PYG, ATCC medium 712) medium (pH 6.5) supplemented with additives (0.05 M CaCl_2_, 0.4 M MgSO_4_·7H_2_O, 0.25 M Na_2_HPO_4_·7H_2_O, 0.25 M KH_2_PO_4_, Na citrate·2 H_2_O, and 0.005 M Fe (NH_4_)_2_(SO_4_)_2_·6H_2_O; Sigma-Aldrich, USA) at 25°C for ~5 days, until a dense monolayer was formed on the bottom surface of the flask. Once optimal growth was achieved, the trophozoite monolayer was washed twice with 5 mL of Page’s amoeba saline (PAS) medium (0.142 g/L Na_2_HPO_4_, 0.136 g/L KH_2_PO_4_, 0.004 g/L MgSO_4_·7H_2_O, 0.004 g/L CaCl_2_·2H_2_O, 0.120 g/L NaCl; Sigma-Aldrich, USA) and harvested by vigorous agitation. The harvested trophozoites were transferred into a Falcon tube and washed twice with DFTW (600 × *g*, 5 min). The final concentration was adjusted to 10^5^ trophozoites/mL using a hemocytometer (Sigma-Aldrich, USA).

### L. pneumophila surrogate preparation and staining.

The solution concentrations and experimental conditions used for the alginate-CaCO_3_ biopolymer surrogate preparation are described in Ariyadasa et al. (16). Briefly, surrogate microparticles with a similar size and shape to those of L. pneumophila were produced via the coprecipitation of CaCl_2_ and low-viscosity alginate. DNA tracer was loaded onto the alginate-CaCO_3_ microparticles using passive adsorption followed by surface modification using poly-l-lysine and poly-l-glutamic acid.

For staining, surrogate microparticles were suspended in 5 mL of 0.22 μm filter-sterilised deionized water and incubated in 15 mL of 1% (wt/vol) alizarin red-S (Sigma-Aldrich, USA), a fluorescent dye which binds the calcium (26) component in the microparticles, for 1 h under continuous stirring at room temperature. ARS-stained microparticles were recovered by centrifugation (1200 × *g*, 5 min). Microparticles were washed twice in filter-sterilised deionized water to remove excess ARS. The pH of the ARS solution was adjusted to 6.5 using 1 M ammonium hydroxide prior to staining the surrogate. DNA tracer was not incorporated into the surrogate microparticles used for visualization experiments, and ARS staining was not performed on the surrogate used in the qPCR studies. ARS emitted brighter fluorescent signals compared to other dyes tested, such as Congo red, methylene blue, and rhodamine 6-G. The ARS-stained microparticles remained fluorescent for up to 2 months after the initial conjugation.

### Cocultures of L. pneumophila*-A. polyphaga* and surrogate-*A. polyphaga* and harvesting trophozoites for qPCR.

Cocultures were established in Cellstar 24-well culture plates following the protocol of Dietersdorfer et al. (27) with modifications. Briefly, a 400 μL trophozoite suspension (10^5^ trophozoites/mL) was transferred into each well of the culture plates and incubated overnight at 30°C to allow for monolayer formation. The monolayer was rinsed with PAS, and stationary-phase L. pneumophila or surrogate (suspended in DFTW) were added into each well at an amoeba to bacteria or surrogate ratio of 1:200. This ratio was selected to facilitate the uptake and detection of the engulfed surrogate. There were 18 L. pneumophila-*A. polyphaga* and surrogate-*A. polyphaga* cocultures established in parallel at 30°C. The engulfment kinetics of each sampling point were analyzed in triplicate. Control samples for each time point consisted of L. pneumophila or surrogate suspended in DFTW in the absence of *A. polyphaga* trophozoites.

Trophozoite monolayers containing engulfed L. pneumophila or surrogate were harvested in duplicate at 0, 2, 4, 6, 12, 24, 36, 48, and 72 h. Each monolayer was thoroughly washed twice with PAS and DFTW prior to harvesting to remove extracellular bacteria and the surrogate. In the L. pneumophila experiments, the washed monolayers were incubated with 50 μg/mL kanamycin sulfate (Gibco, USA) for 1 h at 30°C to kill extracellular bacteria (28). The harvested trophozoites were lysed by freezing at −80°C for 24 h followed by rapid thawing at 37°C to release engulfed L. pneumophila and surrogate for qPCR quantification (29).

### L. pneumophila and surrogate DNA extraction and qPCR.

Prior to DNA extraction, L. pneumophila*-*amoeba cocultures were centrifuged at 5,000 × *g* for 10 min, and the pellet was resuspended in 200 μL of DFTW. In the surrogate-amoeba coculture samples, the entire sample (200 μL) was used for extraction without centrifugation to prevent any surrogate tracer DNA loss during centrifugation. Both the L. pneumophila*-*amoeba and the surrogate-amoeba samples were incubated at 56°C for 1 h in the presence of 180 μL ATL buffer and 20 μL proteinase K. Following incubation, L. pneumophila and surrogate DNA from the control and test samples were extracted using a Qiagen Blood and Tissue extraction kit according to the manufacturers’ instructions.

L. pneumophila and surrogate engulfment were quantified by qPCR (Roche LightCycler 480 real-time PCR system, Roche Applied Science, Germany) using a 70 base-pair (bp) fragment of the *wzm* gene, which is unique to L. pneumophila serogroup 1 (30) and a 200 bp tracer DNA target encapsulated into the surrogate (31), respectively. The *wzm* gene was amplified using the following primer/probe sequences (5′→3′) and thermal cycling conditions: forward primer TGCCTCTGGCTTTGCAGTTA, reverse primer CACACAGGCACAGCAGAAACA, and probe VIC-TTTATTACTCCACTCCAGCGAT-MGBNFQ; thermal profile: 1 cycle at 95°C for 5 min, followed by 45 cycles at 95°C for 15 sec and 60°C for 1 min (30). The thermal profile used for surrogate tracer DNA amplification was 1 cycle at 95°C for 10 min, 45 cycles at 95°C for 10 sec, 60°C for 30 sec, and 72°C for 12 sec.

The L. pneumophila standard curve used in the experiments was established using 8 triplicate L. pneumophila oligomers, which ranged from 60 to 6 × 10^8^ DNA copies per qPCR, with an amplification efficiency of 89.09%, an amplification factor of 1.89, and a detection limit of 14 DNA copies/5 μL. Similarly, the surrogate tracer DNA standard curve was generated using 7 triplicate tracer DNA oligomers between concentrations of 2 to 2 × 10^9^ DNA copies per qPCR with an amplification efficiency of 98%, an amplification factor of 1.98, and a detection limit of 5 DNA copies/4 μL. The concentrations of the intracellular surrogate are reported in copies/4 μL, and the concentrations of the bacteria are reported in copies/5 μL. Normalized concentrations were used to compare the engulfment kinetics.

### Plate counts of engulfed L. pneumophila.

L. pneumophila engulfed by trophozoites at each sampling point were enumerated by adapting a method published by Conza et al. (32). Briefly, 40 μL of the harvested L. pneumophila*-*amoeba coculture samples were diluted 1:10 using 0.2 M HCl-KCl buffer (pH 2.2) and incubated for 10 min at room temperature to rupture the trophozoites. The acid lysis method was selected for rupturing the trophozoite monolayers in the L. pneumophila enumeration experiments, as 0.2 M HCl-KCl does not affect the culturability of the bacteria, as opposed to the freeze-thaw method used in the qPCR quantification studies. The suspensions were vortexed 3 times during the incubation period, serially diluted (10^0^, 10^−1^, and 10^−2^), and plated on BCYE-GVPC agar. Plates were incubated at 37°C for 5 days for colonies to appear. When plating the control samples, 100 μL each of the serially diluted L. pneumophila in DFTW (10^−5^ and 10^−6^) were directly plated on BCYE-GVPC agar plates.

### Surrogate*-A. polyphaga* coculture, CLSM, and image analysis.

Surrogate engulfment by *A. polyphaga* trophozoites was visualized using a TCS SP5 confocal laser scanning microscope (Leica Microsystems, Germany). Prior to coincubating the trophozoites with the ARS-stained surrogate, 400 μL of a 10^5^ trophozoites/mL suspension was transferred into a cell culture dish (FluoroDish, World Precision Instruments, USA) and incubated overnight at 30°C to form a monolayer. After incubation, the nonadherent trophozoites were removed via the careful aspiration of the supernatant, and the monolayer was rinsed twice with 400 μL of PAS. Subsequently, 400 μL 2 × 10^7^ microparticles/mL of ARS-stained surrogate suspended in DFTW were added to the washed monolayer at an amoeba to surrogate ratio of 1:200. CLSM time-lapse images of the surrogate-*A. polyphaga* coculture were obtained every 15 sec at 0, 3.5, 12, 24, and 48 h of coculture with the DPSS561 laser (at 60% laser power) in the visible spectrum, using the xyt scanning mode to observe the progression of the surrogate engulfment. The imaging parameters used in the acquisition were as follows: format, 1024 × 1024; speed, 400 Hz; image size, 101.93 μm × 101.93 μm; pixel size, 99.64 nm × 99.64 nm; zoom factor, 1.4; and rotation, 0. Raw Leica image file format (.lif) data files generated from the CLSM imaging were processed using the Bio Formats Importer plugin of the ImageJ software (33). To process the images, the CLSM data files were first imported using the Bio Formats importer plugin to covert the metadata acquired through the microscope into an ImageJ readable format. Image stacks were viewed in hyperstack mode with the default color option in 8-bit color. The “spilt channels” option was used to view bright field and fluorescence images, whereas the “merge channels” option was used to obtain composite images. A size analysis macro (Appendix 1 in the supplemental material) was used to enumerate and derive the surface areas of the extracellular surrogate microparticles. The experiments were conducted in duplicate. The control samples for each time point consisted of *A. polyphaga* trophozoites suspended in DFTW in the absence of fluorescent surrogate.

### Statistics.

The surrogate and L. pneumophila engulfment kinetics of *A. polyphaga* are represented as the average ± standard deviation of 6 sample replicates from 2 experiments. 20 CLSM fields of view from 2 individual experiments were used in the enumeration and surface area analysis of the extracellular surrogate. 10 and 8 fields of view were enumerated at each time point to determine the number of free surrogates per field and the ratio of active to rounded trophozoites, respectively. Statistical significance was assessed using a paired Student's *t* test.
