# Supplementary material for: Use of a Novel DNA-Loaded Alginate-Calcium Carbonate Biopolymer Surrogate to Study the Engulfment of Legionella pneumophila by Acanthamoeba polyphaga in Water Systems
Source: Microbiol Spectr. 2022 Aug 11;10(4):e02210-22. doi: 10.1128/spectrum.02210-22 (PMC9430812; doi:10.1128/spectrum.02210-22)
Supplement: Supplemental file 1 — Supplemental material. Download spectrum.02210-22-s0001.pdf, PDF file, 0.4 MB [file spectrum.02210-22-s0001.pdf]

Supplementary Material

Use of a novel DNA-loaded alginate-calcium carbonate biopolymer surrogate to study engulfment of *Legionella pneumophila* by *Acanthamoeba polyphaga*

Sujani Ariyadasa[1,2], Craig Billington[1#], Mohamed Shaheen[3], Nicholas J. Ashbolt[4], Conan Fee[5], Liping Pang[1]

[1] Institute of Environmental Science and Research, PO Box 29181, Christchurch 8540, New Zealand.

[2] School of Biological Sciences, University of Canterbury, Private Bag 4800, Christchurch 8041, New Zealand.

[3] School of Public Health, University of Alberta, Edmonton T6G 2G7, Canada

[4] Faculty of Science & Engineering, Southern Cross University, PO Box 157, Lismore, NSW, 2480, Australia.

[5] School of Product Design and Biomolecular Interaction Centre, University of Canterbury, Private Bag 4800, Christchurch 8041, New Zealand.

[#] Corresponding author.

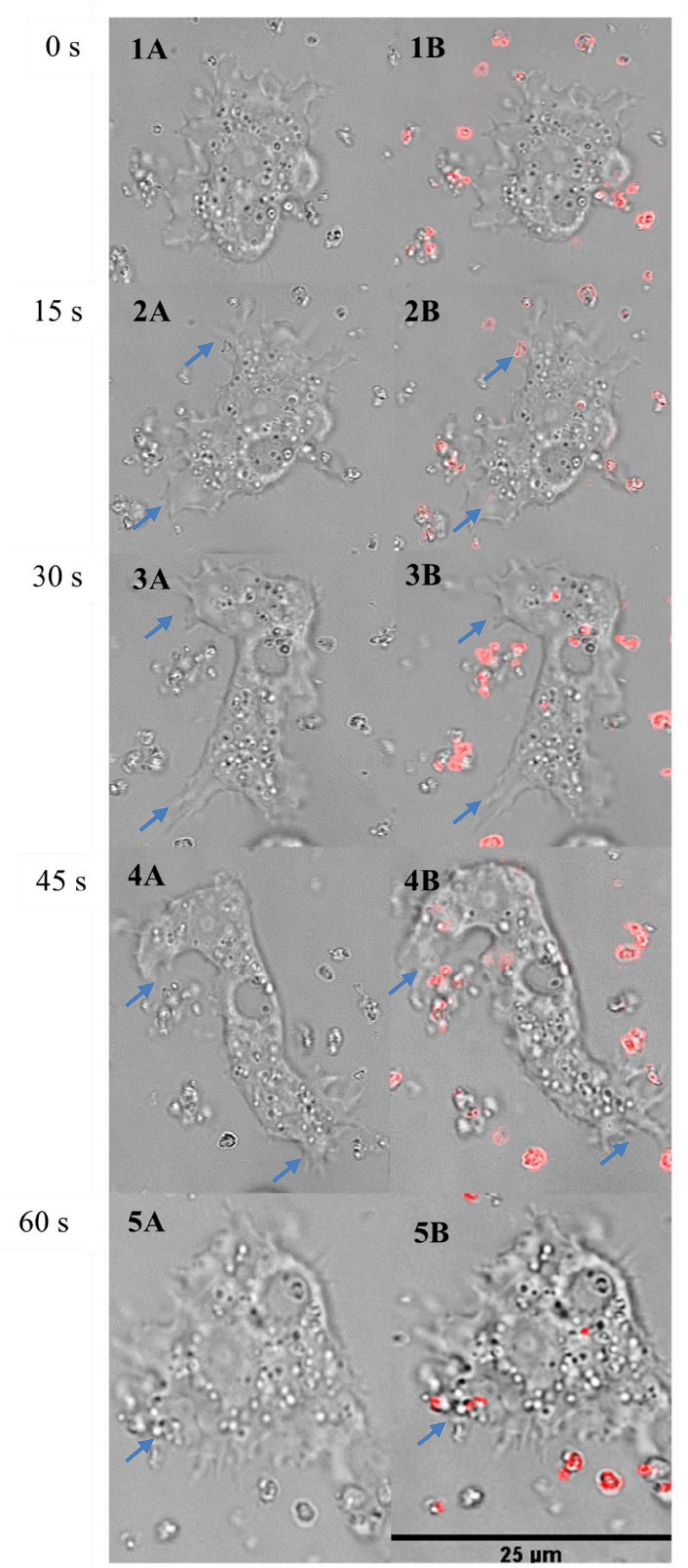

Figure S1. CLSM bright-field (1A-5A) and composite (1B-5B) time-lapse images of *A. polyphaga* acanthopodia formation and surrogate engulfment immediately after co-culture establishment at 100×. Blue arrows point to *A. polyphaga* acanthopodia.

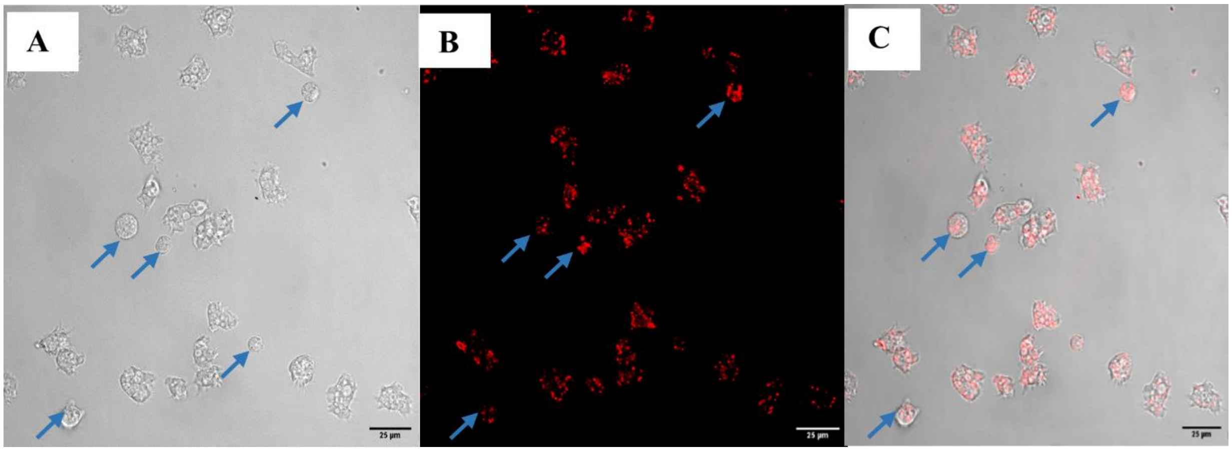

Figure S2. CLSM bright-field (A), fluorescent (B), and composite (C) images of surrogate-*A. polyphaga* co-cultures at 12 h at 45×. Blue arrows point to rounded *A. polyphaga* trophozoites with retracted acanthopodia.

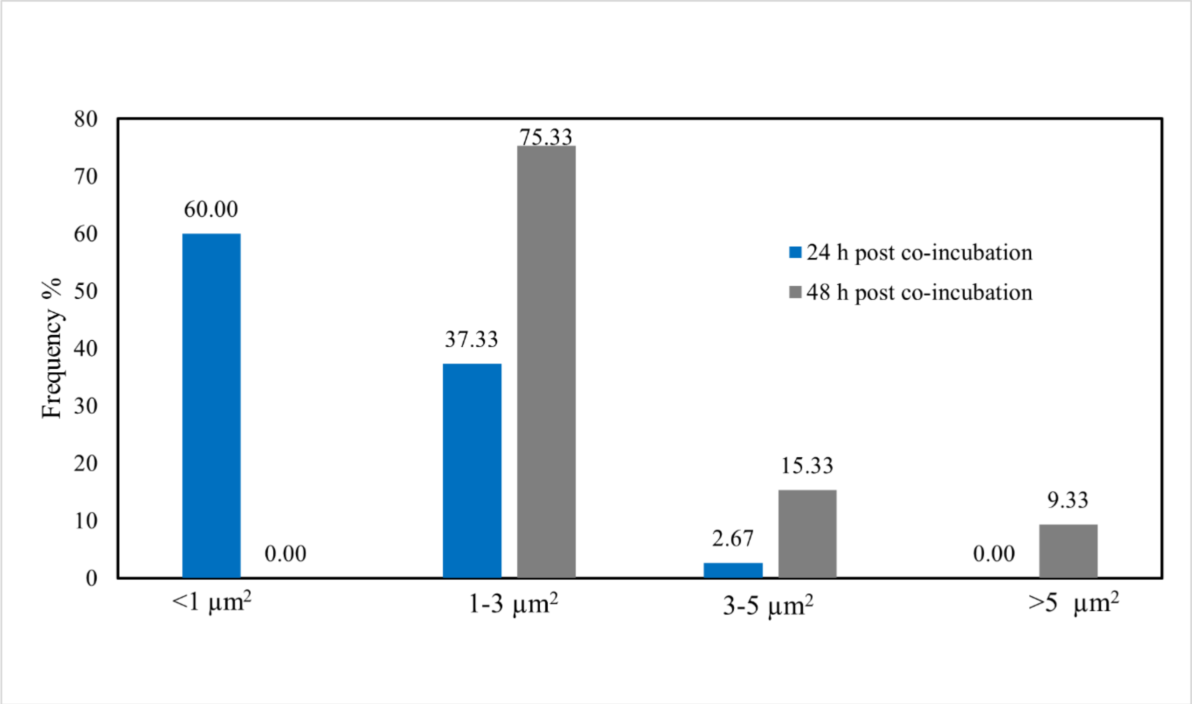

Figure S3. Size distribution of surrogate clumps released by *A. polyphaga* trophozoites at 24 h and 48 h following co-incubation at 30 °C.

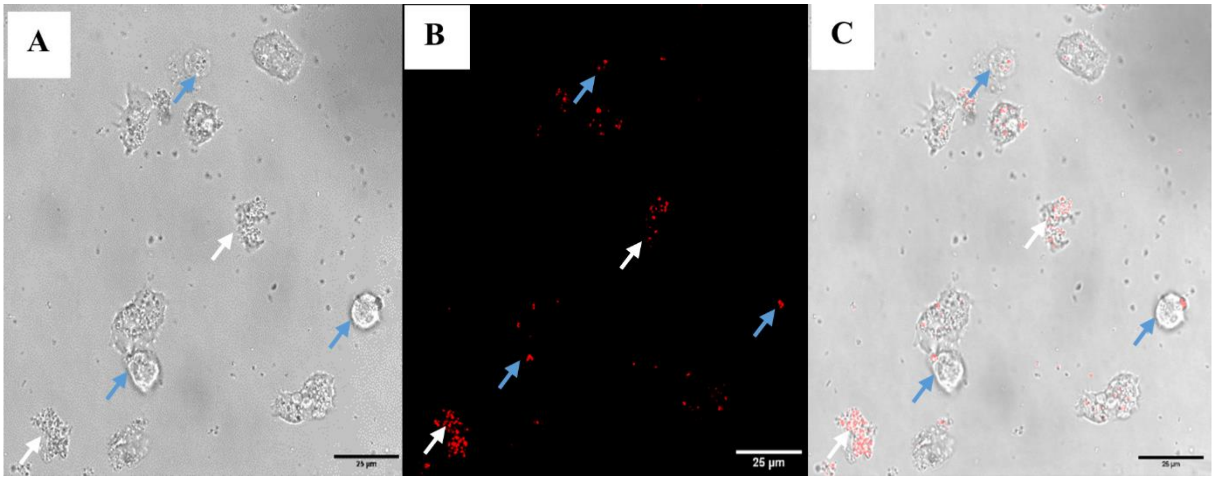

Figure S4. CLSM 60× bright-field (A), fluorescent (B), and composite (C) images of surrogate-*A. polyphaga* co-cultures at 48 h. White and blue arrows point at lysed trophozoites and round trophozoites with retracted acanthopodia, respectively. Magnification 45×.

Table S1. Counts of active (with acanthopodia), round, and lysed trophozoites per microscopy field at 12 and 48 h after inoculation of surrogates. Images obtained at 45x.

hours after inoculation of surrogates	number of trophozoites w/ acanthopodia per field	number of round trophozoites per field	number of lysed trophozoites per field	active to round trophozoite ratio per field	average	std dev	T.test
12	17	4	0	4.3	5.0	1.6	
12	21	6	0	3.5			
12	19	3	0	6.3			
12	22	3	0	7.3			
12	14	3	0	4.7			
12	16	4	0	4.0			
12	22	7	0	3.1			
12	20	3	0	6.7			
							0.601
48	15	1	3	15	13.3	3.6	
48	16	1	2	16			
48	13	0	4	0			
48	13	1	4	0			
48	15	0	2	0			
48	14	0	0	0			
48	14	1	3	14			
48	16	2	3	8			
